# Timepix3: Temperature Influence on Radiation Energy Measurement with Si Sensor

**DOI:** 10.3390/s23042201

**Published:** 2023-02-15

**Authors:** Martin Urban, Ondrej Nentvich, Lukas Marek, Rene Hudec, Ladislav Sieger

**Affiliations:** 1 Faculty of Electrical Engineering, Czech Technical University in Prague, Technicka 2, 166 27 Prague 6, Czech Republic; 2 Faculty of Mathematics and Physics, Charles University, V Holesovickach 2, 180 00 Prague 8, Czech Republic; 3 Advacam, s.r.o., U Pergamenky 1145/12, 170 00 Prague 7 , Czech Republic

**Keywords:** Timepix3, X-ray detector, energy measurement, temperature effects, compensations

## Abstract

The Timepix3 readout ASIC chip is a hybrid pixelated radiation detector, designed at CERN, which contains a 256 px × 256 px matrix. Each of the 65,536 radiation-sensitive pixels can record an incoming particle, its energy deposition or time of arrival and measure them simultaneously. Since the detector is suitable for a wide range of applications from particle physics, national security and medicine to space science, it can be used in a wide range of temperatures. Until now, it has to be calibrated every time to the operating point of the application. This paper studies the possibility of energy measurement with Timepix3 equipped with a 500 m thick silicon sensor and MiniPIX readout interface in the temperatures between 10 ${}^{\circ }C$ and 70 ${}^{\circ }C$ with only one calibration. The detector has been irradiated by X-ray fluorescence photons in the energy range from 8 keV to 57 keV, and 31 keV to 81 keV photons from the ^133^Ba radioactive source. A deviation of 5% in apparent energy value may occur for a 10 ${}^{\circ }C$ change in temperature from the reference point, but, with the next temperature change, it can reach up to −30%. Moreover, Barium photons with an energy of 81 keV appear as deposited energy of only 55 keV at a detector temperature of 70 ${}^{\circ }C$. An original compensation method that reduces the relative measurement error from −30% to less than 1% is presented in this paper.

## 1. Introduction

Due to the Timepix3 (TPX3) hybrid structure, these detectors may be used in a wide range of scientific and industrial fields. Detectors from the Medipix and Timepix family are often used for X-ray imaging [1], dosimetry, or as an environmental radiation monitor. The detectors’ high-resolution imaging capabilities are applicable to the analysis of paintings and sculptures [2,3] as well as mammography [4], dental imaging [5] and CT scans [6]. Significant applications include X-ray inspection of material joints and faults and the localisation of radiation sources [7,8]. The TPX3 detector, in combination with a suitable converter, can also be used for neutron detection of both D-D and D-T sources. This has been demonstrated in previous research studies [9,10], which have shown its effectiveness in detecting both fast and thermal neutrons.

The predecessors of TPX3 were used in scientific instruments such as ATLAS [11], the suborbital rocket campaign [12,13,14] and the following satellites: PROBA-V with SATRAM experiment [15,16,17]; British TechDemoSat-1 with LUCID experiment [18]; Czech CubeSat VZLUSAT-1 [19,20,21] and RISEPix on the RISESAT microsatellite [22].

In many applications, the detectors undergo thermal changes in the environment and thus in the detector’s structure. Temperature stability and known dependence of device behaviour on temperature are important in the case of the TPX3 detector as well as in the case of most modern semiconductor sensors.

The accuracy of the resulting energy measurements is closely related to the conditions under which the measurement was made or their likeness to the conditions under which the detector was calibrated. For this reason, several calibration methods have already been described, e.g., using X-ray fluorescence (XRF) and low-energy gamma rays radiation sources [23], internal test pulses [24,25], protons [26,27], or alpha particles [28,29].

Since the detector can be used in many applications and in various environments, significant changes in operating temperatures of up to tens of degrees Celsius can occur. Achieving standard measurement conditions (identical to calibration conditions) can be challenging. Temperature stabilisation in the target application can also be very power-demanding, especially for space usage. The characterisation of the detector and the description of its temperature dependencies are essential to minimising the distortion of the measured data.

Many articles mention the possible applications of TPX3; however, there are only a few papers that describe the characterisation of the detector in a variable environment or on measurement mode [30,31]. This manuscript presents the results of temperature tests during radiation energy measurements performed with the MiniPIX TPX3 detector. The tests were conducted over a wide operating temperature range from 10 ${}^{\circ }C$–70${}^{\circ }C$. The characterisation was realised using XRF and a radiation source in the energy range from 8 keV to 81 keV. Based on the observed dependencies, a linear correction of the measured energy deviation was proposed and presented in the final part of this paper.

The remaining part of the article is divided into several sections. Section 2 contains a brief description of the detector and the detection principle. Next, Section 3 describes the setup, materials, radiation sources and methods used. Section 4 presents the detector’s temperature dependence and the effect of temperature drift on the acquired energy spectra using several parameters, such as the accuracy of the measurement. Section 5 presents and proposes a novel method for linear correction of the detected temperature distortion of the energy spectra. Section 6 is devoted to a final summary of the observed phenomena, properties and presented methods.

## 2. Detector Description

The TPX3 is a hybrid active pixel detector with a semiconductor detection layer. This detection chip was developed at CERN as a successor to the Timepix chip [32] in the Medipix collaboration. The TPX3 is a Complementary Metal–Oxide–Semiconductor (CMOS) type of detector with a square matrix of 256 px × 256 px and a pixel pitch of 55 μm [33]. The device contains a readout Application-Specific Integrated Circuit (ASIC) chip on which a semiconductor sensor layer is bump-bonded. Within the ASIC, there are integrated electronics to sense and evaluate the signal from each pixel. The energy threshold discriminator, integrated in ASIC, is used to eliminate noise (identified as low energy events); therefore, it is possible to generate a noise-free system when the calibration and equalisation are performed.

The ASIC allows independent reading of individual pixels in data-driven readout mode, where only the hit pixels are read out continuously during the exposure time. In this readout mode, the detector can have virtually zero dead time, but individual pixels still have about 475 ns dead/readout time, extended with time for the discharging of accumulated charge.

The TPX3 ASIC can operate all the 65536 px in one of the following modes:

Time-of-Arrival (ToA) mode —measurement of the particle impact time to the detector. That is, the time it takes to reach the Threshold (THL) level from the beginning of the exposure time.

Time-over-Threshold (ToT) and ToA mode—combination of simultaneous ToA and deposited energy measurements. The measurement of the deposited energy in a pixel from the incident particle is based on the Wilkinson-type Analogue-to-Digital Converter (ADC). The sensed energy depends on the number of clock ticks during which the signal is above the preset discrimination level (Threshold). The Time-over-Threshold relies on the time (number of clock ticks) it takes to discharge the accumulated charge in each pixel by a constant current.

Event counting and integral ToT mode—the count of events and their integrated charge in each pixel during the common exposure time. Only events generating the charge above the discrimination level are sensed.

**Figure 1:**
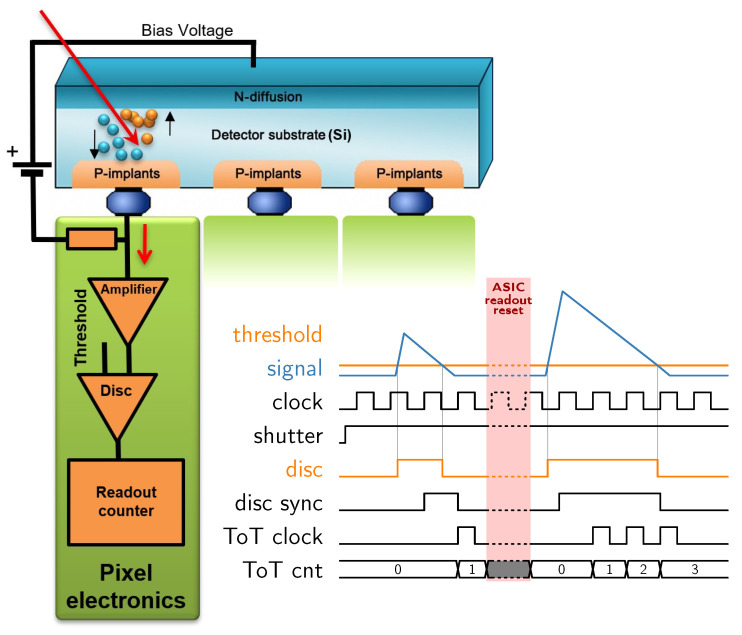
Schematic representation of Timepix3 detector function in Time-over-Threshold measurement.

This paper examines the effect of temperature on the measurement of deposited energy in the ToT and ToA mode via data-driven readout. A simplified diagram of the measurement principle is shown in Figure 1.

One of the advantages of Timepix3 operated in the data-driven readout mode is the possibility of acquiring complete information about the interaction of the particles immediately after its impact to the detector [33]. Within the collected data stream, the ToA, ToT values and pixel coordinate information are available for each interaction in the presented case. The detection layer for the Medipix/Timepix detector family can be made of different semiconducting or semi-insulating materials, such as Si, GaAs, CdTe, CZT, etc., in combination with the same ASIC chip due to their hybrid structure. The monolithic sensing layer can have various thicknesses, typically ranging from 100 μm to 2000 μm, depending on the application and its energy detection range, but the thickness within the sensor is constant with respect to manufacturing precision.

## 3. Measurement and Methods

**Figure 2:**
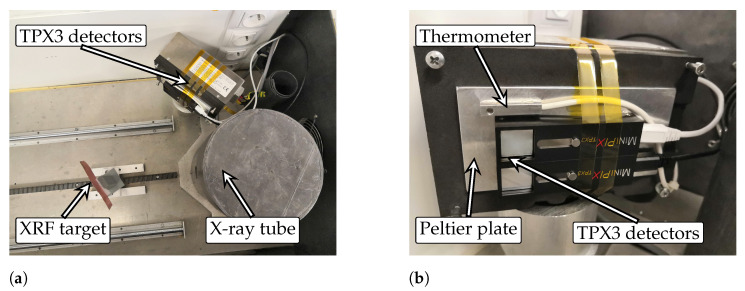
Detector arrangement for thermal testing of the Timepix3 detectors. (**a**) arrangement configuration for X-ray fluorescence measurement; (**b**) details of the detector placement on the Peltier module with feedback thermometer.

The data presented in this paper were collected during measurements with three MiniPIX TPX3 detectors equipped with a 500 μm Si sensor with 200 V bias voltage applied. The detectors were thermally coupled to the Peltier plate using a thermally conductive tape between the detector and the cold/heat plate, and the whole mounting was secured by Kapton tape. Temperature stabilisation of the cold/heat plate was conducted at several temperatures in a range from 10 ${}^{\circ }C$ to 70 ${}^{\circ }C$ with 10 ${}^{\circ }C$ steps. This temperature range is suitable for testing radiation detectors as it covers a wide range of temperatures representative of different environments and hence a significant part of their applications such as laboratory, medical, industrial, outdoor and some applications in space. The temperature of the cold/heat plate was controlled and monitored by a feedback temperature sensor. Since the thermometers provided by the manufacturer in the Timepix3 ASIC are uncalibrated, the sensed values throughout the tested detectors were inaccurate by several degrees of Celsius. Therefore, an external thermometer connected by the four-wire method was chosen for temperature measurement, which was thermally coupled to minimise the potential temperature gradient. After reaching the target temperature, the detectors were temperature stabilised for at least 10 min before each set of measurements was performed. Each measurement set contains a series of energy spectrum measurements in the ToT and ToA data-driven mode for individual sources of radiation. During the measurements, the setup was placed in a shielded X-ray box equipped with an X-ray tube and holder for X-ray fluorescence targets. The arrangement of the experiment, including details of the measurement setup, is shown in Figure 2.

Four materials (Cu, Mo, In, Ta) were selected as targets for the generation of the characteristic radiation generated by X-ray fluorescence. The advantage of XRF is its precise definability of the individual spectral lines. The fifth (last) tested source is a radioisotope of Barium which adds spectral peaks in the tested energy range. All used sources of radiation and their characteristic energies are listed in Table 1.

**Figure 3:**
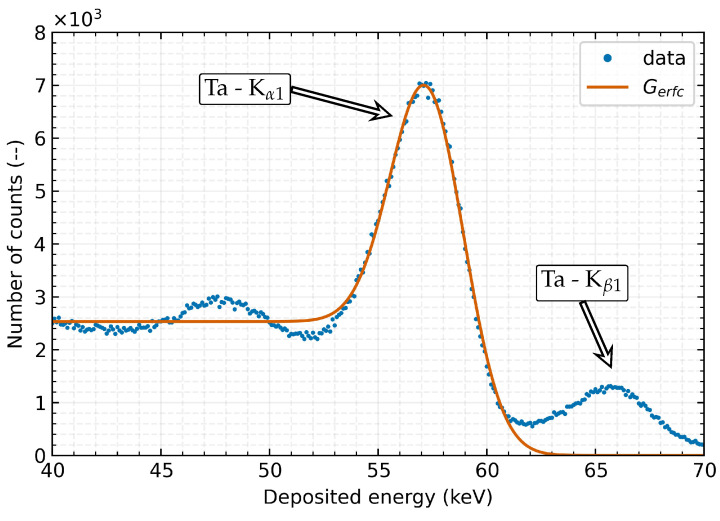
Measured spectral peak generated by the X-ray fluorescence of a tantalum target. The measured data (blue line), together with the result of the applied $G_{erfc}$ function fit on the Ta-K${}_{\alpha 1}$ XRF peak (orange line), are shown on the graph. The TPX3 detector with a 500 μm Si detection layer was stabilised at 30 ${}^{\circ }C$ throughout the measurement.

With the usage of a well-defined radiation source, it is possible to measure and determine changes in the detector’s response to clearly defined stimuli and thus establish its dependence on external conditions such as temperature. The spectral peaks (Figure 3) are fitted by a $G_{erfc(x;A_{1},A_{2},\mu ,\sigma )}$ (Equation (1)) function which is a combination of Gaussian $G_{\left(x;A_{1},\mu ,\sigma \right)}$ (Equation (2)), which represents radiation peak, with complementary error function $E_{rfc}\left(x\right)$ (Equation (3)) as a representation of decreasing characteristics of the measured energy spectrum. This approximation is used to determine the position of the peak centre μ and σ as its standard deviation. The *x* is the value of deposited energy:

(1) $$G_{erfc(x;A_{1},A_{2},\mu ,\sigma )}=G_{\left(x;A_{1},\mu ,\sigma \right)}+A_{2}\;\cdot \;E_{rfc}\left(\frac{x-\mu }{\sigma \sqrt{2}}\right)$$

where

(2) $$G_{\left(x;A_{1},\mu ,\sigma \right)}=A_{1}\;\cdot \;e^{\frac{-{\left(x-\mu \right)}^{2}}{2\sigma^{2}}}$$

and

(3) $$E_{rfc}\left(x\right)=1-\frac{2}{\sqrt{\pi }}\int_{0}^{x}e^{-t^{2}}dt.$$

The procedure for obtaining the measured energy spectra includes clustering of each event, which takes into account multi-pixel clusters and reconstructs their original energy. The examined detectors were calibrated as part of the standard manufacturing procedure before the characterisation starts. The calibration procedure consists of several steps such as threshold equalisation, which compensates for non-uniformities within the pixel matrix. This is followed by a global calibration of the threshold energy, which consists of a measurement series depending on the sequential change of the applied threshold for several monochromatic radiation energies, and Time-over-Threshold calibration for per pixel energy measurement. The THL calibration procedure is described in Urban and Doubravová [31] as well as ToT energy calibration in Jakubek [23].

## 4. Thermal Dependency

This chapter describes some of the main parameters such as *Absolute measurement accuracy*, *Relative error of measurement*, and *Relative energy resolution* of the Timepix3 detectors equipped with a Si sensor and their temperature dependence. Several sets of measurements were used for the evaluation. Temperature fluctuations during the measurement affect the detector output and distort the evaluated data. The used data sets include measurements of energy spectra from XRF sources as well as from natural sources (see Table 1), and all tested detectors were sequentially thermally stabilised in several steps ( 10 ${}^{\circ }C$, 20 ${}^{\circ }C$, …, 70 ${}^{\circ }C$).

**Figure 4:**
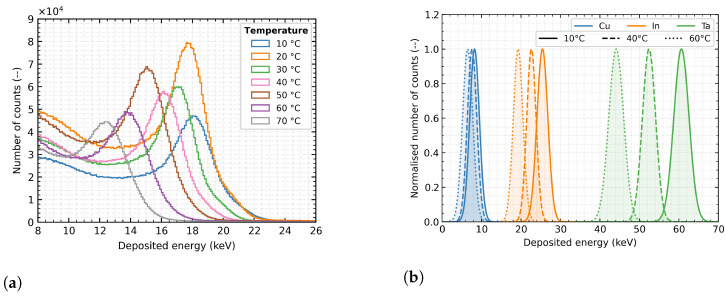
The thermal influence on energy measurement. (**a**) the set of spectral measurements depends on the detector temperature from 10 ${}^{\circ }C$–70 ${}^{\circ }C$ for constant settings of the X-ray tube with the Molybdenum XRF target; (**b**) visualisation of the temperature effect on the measured incident radiation energy. Normalised Gaussian curves represent the individual spectral peaks for better clarity. The detector was calibrated for 20 ${}^{\circ }C$.

The measured set for one detector, one measured energy (same XRF target) with constant X-ray tube setting and different temperatures is shown in Figure 4a. For a more explicit presentation of the thermal effect on the accuracy of the energy measurement at different energies, see Figure 4b. For clarity, the individual spectral peaks are represented by a normalised Gaussian curve, making recognising the resulting distortion in the energy spectrum clearer.

According to the manufacturer, the most common calibration of these detectors is performed at a temperature of 20 ${}^{\circ }C$. Therefore, the measurements at this temperature are used as a reference for the subsequent evaluation. The numerical subscripts given in the formulas below are used as a reference for the measurement temperature.

### 4.1. Absolute Measurement Accuracy

The first evaluated parameter is the absolute measurement accuracy, which is defined as the absolute shift of the measured energy of the incident radiation (Equation (4)), where $\mu_{x}$ is the position of the measured peak centre at temperature *x* and $\mu_{20}$ is the mean position at 20 ${}^{\circ }C$:

(4) $$\Delta \mu =\mu_{x}-\mu_{20}$$

**Figure 5:**
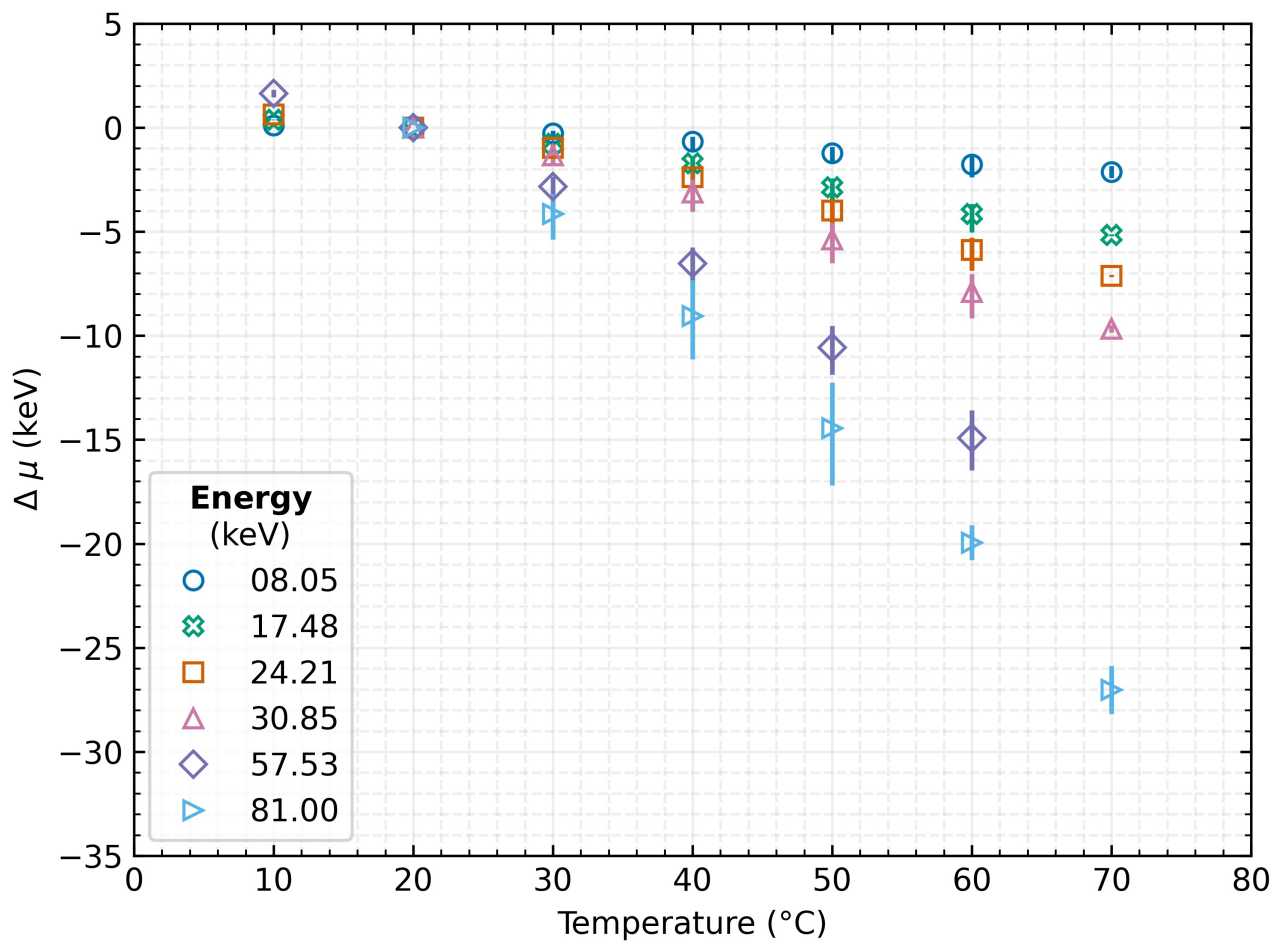
Absolute shift of the spectral peak for a given energy with respect to the detector’s temperature. The data represent the mean values over the tested detectors, and the error bars indicate their minimum and maximum value.

The results indicate that, with increasing detector temperature, the sensed energy shifts toward lower values. Thus, the accuracy of the measurement decreases, and this effect is more significant as the energy of the incident radiation increases (see Figure 5). As can be seen, when the detector was heated up to 70 ${}^{\circ }C$ from the reference 20 ${}^{\circ }C$, the measured energy peaks were shifted by −2.10 keV at 8.05 keV, −9.67 keV at 30.85 keV, and −27 keV at 81 keV.

### 4.2. Relative Error of Measurement

Following the change in the absolute accuracy of the measurements, the relative percentage error of the measurements for the individual energies was also evaluated:

(5) $$RE_{20}=\frac{\mu_{x}-\mu_{20}}{\mu_{20}}\;\cdot \;100\%$$

**Figure 6:**
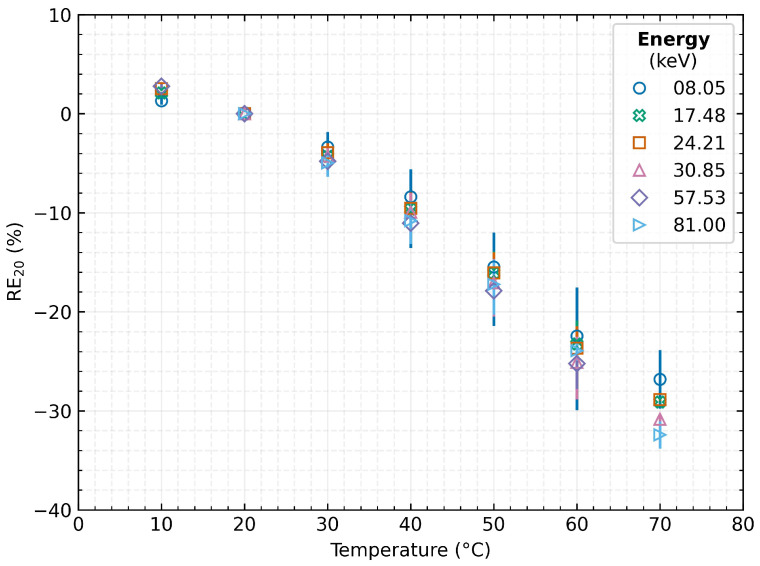
Dependence of the relative measurement error on the detector temperature for different energies of incident radiation. The plotted data represent the mean values over the tested detectors, and the error bars indicate their minimum and maximum value.

This evaluation (see Figure 6) led to the finding that the resulting accuracy of the measurement is not as significantly dependent on the energy of the incident radiation as it might appear from Figure 5. However, the energy dependence still persists, and the temperature change has a more significant effect at higher energies (for 30 ${}^{\circ }C$−3.36% at 8.0 keV vs. −4.79% at 57 keV). Therefore, the relative error of the measurement is within 5% when the detector is warmed by 10 ${}^{\circ }C$. However, as the temperature rises, the distortion of the detected peak increases as well, up to −26.8% at 8.0 keV and −32.4% at 81 keV at 70 ${}^{\circ }C$. According to the results presented in this section, it is evident that the percentage error in measurement accuracy increases rapidly with rising detector temperature and reaches 30% when a sensor is heated to 70 ${}^{\circ }C$.

### 4.3. Relative Energy Resolution

Finally, the energy resolution was evaluated according to Equation (6) as well as its dependence on temperature. The definition of energy resolution implies that the lower the percentage value of the quantity, the better the resulting resolution. For the calculation, the accurate material energy *E* and evaluated Full Width at Half Maximum (FWHM) of the measured peak is always used from each measurement set. For the purpose of this evaluation, the FWHM is defined as $FWHM_{x}=2\sqrt{2\ln2}\sigma_{x}$, i.e., $\approx 2.35\sigma_{x}$. The subscript *x* represents, the same as in the previous cases, the temperature corresponding to the particular measurement:

(6) $$Re=\frac{FWHM_{x}}{E}\;\cdot \;100\%$$

**Figure 7:**
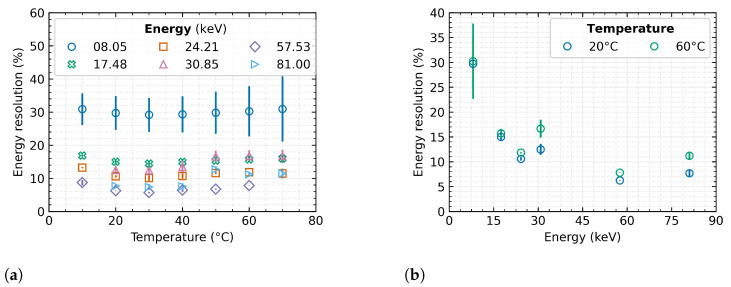
The energy resolution of Timepix3 detector with 500 μm Si sensor. The plotted data represent the mean values over the tested detectors, and the error bars indicate their minimum and maximum value. (**a**) dependence on detector temperature; (**b**) dependence on radiation energy.

The characteristics in Figure 7a represent the dependence of energy resolution on sensor temperature for several radiation sources.

In the range from 20 ${}^{\circ }C$–40 ${}^{\circ }C$, there is only a minor effect of the sensor temperature on the spectral resolution of the measured signal. Change of the energy resolution is within 0.5% over the entire tested spectral range. As the temperature increases, the spectral peaks widen, so the energy resolution gradually decreases.

Nevertheless, the energy resolution improves significantly with increasing radiation energy (Figure 7b), which is consistent with the theoretical assumptions and confirms measurements in Nowak et al. [30]. This figure is also expanded by comparison of the trends at two measured temperatures, where there is a clear tendency of declining spectral resolution with dependence on increasing detector temperature.

## 5. Linear Compensation

Based on the presented energy dependencies of the MiniPIX TPX3 detector, a global correction of the energy spectra can be proposed. The correction method is based on the knowledge of the measurement accuracy evolution depending on the detector temperature for individual incident radiation energies (see Section 4.1).

**Figure 8:**
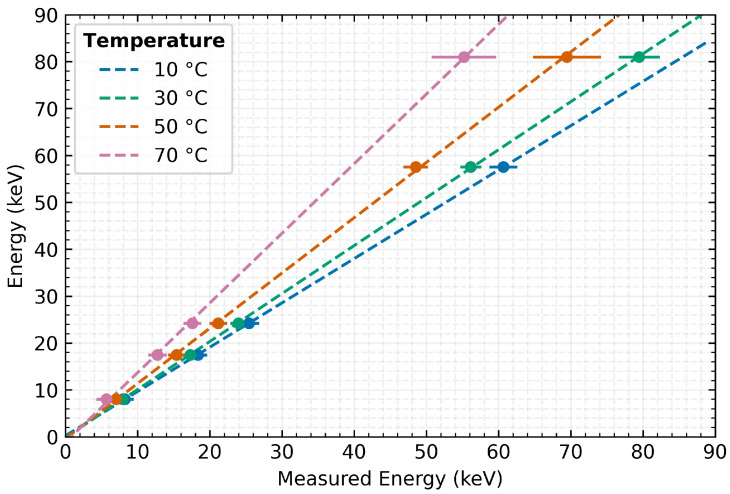
The correction functions for the global energy spectra of one of the tested detectors. Error bars represent the standard deviation of the measured value of the energy maximum.

As can be seen in Figure 8, a linear function can be established for different stable temperatures of operation. A control measurement was performed to validate the proposed method with the detector stabilised at 40 ${}^{\circ }C$ and 70 ${}^{\circ }C$ and using the ^152^Eu radionuclide ( 39.91 keV) as the radiation source. This radiation source is not included in the data set of measurements based on which the correction function was determined.

**Figure 9:**
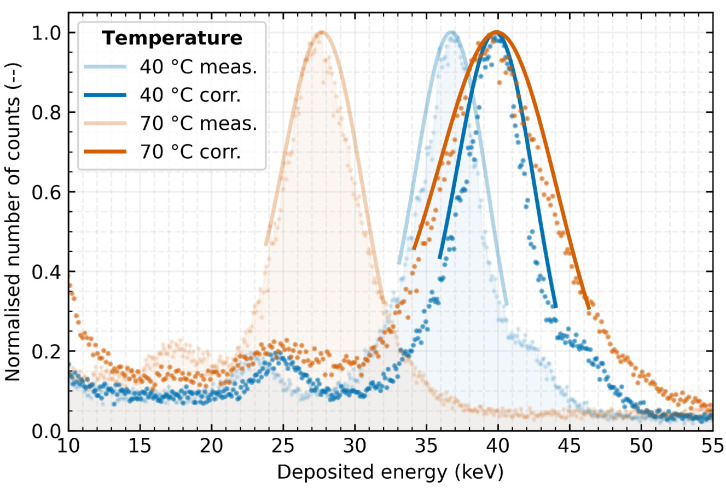
Measurement of the Europium radionuclide energy spectrum with the detector at 40 ${}^{\circ }C$ and 70 ${}^{\circ }C$. Comparison of measured energy spectra before (light curves) and after linear correction (dark curves) according to dependencies in Figure 8.

Figure 9 clearly shows the influence of measuring the same radiation source at different temperatures and indicates the effect of the correction on the measured spectra. The same already presented fit model as in the previous cases is used for the evaluation. The results show that the application of the correction successfully compensates for the temperature distortion of the measurement accuracy. Applying the proposed linear correction method to the measured data improved the absolute measurement accuracy error from −3.07 keV to 0.04 keV for 40 ${}^{\circ }C$ detector temperature and from −11.99 keV to 0.28 keV for 70 ${}^{\circ }C$. Corresponding to these changes, the relative measurement error decreases from −47.7% and −30.0% to 0.1% and 0.7%, respectively. However, even after the application of the correction, the energy resolution is degraded by rising detector temperature causing an increasing FWHM of the spectral peak. In the above case, the change in resolution (after correction) is from 16% at 40 ${}^{\circ }C$ to 24% at 70 ${}^{\circ }C$. The obtained energy spectrum at the validation temperatures corresponds persistently to the radiation energy of the applied radiation source.

## 6. Conclusions

This manuscript presents the results of the Time-over-Threshold response characterisation of Timepix3 detectors with the MiniPIX readout interface, exposed to various temperature and radiation conditions. The tested detectors were equipped with a 500 μm thick silicon sensor and stabilised at various temperatures in the range from 10 ${}^{\circ }C$–70 ${}^{\circ }C$, where they were exposed to X-ray radiation in the energy range from 8 keV to 81 keV.

Based on the performed experiments, it was found that the distortion of the measured signal does not depend only on the detector temperature but also on the energy of the incident radiation. It was observed that, as the temperature increases, the measured energy spectrum systematically drifts towards lower energy values of sensed energy. The absolute energy shift is dependent on the energy value of the incident particles and becomes more significant as their energy increases. This shift can cause an absolute error of several tens of kilo-electron volts, which means a relative measurement error of up to −30% in the tested energy/temperature set.

According to Mazziotta [34], the temperature dependence of the electron–hole pair creation in the presented temperature range was found to be at most 0.55%. This suggests that the change in the pair creation energy has a minimal impact.

Analysis of the energy resolution, which provides information about the detector’s ability to distinguish two close spectral lines, shows that the energy resolution strongly depends on the incident energy. As the value of relative resolution decreases, the ability to recognise tight spectral lines from each other grows as the energy of the impacting particles increases. Based on the analysis, it can be noticed that the detected deviation of the energy resolution over the tested X-ray fluorescence energies does not exceed 2% for temperatures from 20 ${}^{\circ }C$–40 ${}^{\circ }C$, respectively 5% over the whole range of tested temperatures. Therefore, this kind of detector proves significant spectral resolution stability over a 20 ${}^{\circ }C$ temperature range, with only minor changes.

Previous studies indicate that an increase in temperature difference, both positive and negative, can result in a disruption of the homogeneity of the equalisation matrix [35,36] because of differences in leakage current in each pixel and its effect on electronics, leading to a non-uniform distribution of pixel noise throughout the full frame and possibly a widening of peaks in the energy spectrum.

Other important elements that can affect energy measurement are noise sources. The most significant noise sources include shot noise, thermal noise and flicker noise. In dependence on the type and the noise generation method, its intensity and other characteristic properties may depend on the device’s temperature [37,38,39].

To the best of our knowledge, this paper is the first which describes the temperature dependence of the energy measurement using the Timpeix3 detector and, based on its characterisations, is the first in the literature to propose and describe the correction method for the constant offset from the calibration temperature. The fast and efficient correction method uses the observed linear dependence of the measurement error. The presented method allows for reducing the degradation of the absolute accuracy of the measurement below 0.5 keV. For the validation measurement on the ^152^Eu sample, which was not included in the modelling set, a reduction of the relative measurement error to 0.7% from the original −30% was achieved with this method. However, there is still a degradation of FWHM with increasing temperature.

Based on the presented results and the fact that mainly the position of each energy peak changes (change of the measurement accuracy), it can be inferred that the primary source of measurement variation is the electronics and the ASIC chip rather than the semiconductor sensor itself. The characteristics of the observed detector’s distortion suggest that the change in temperature may cause a shift in the threshold or a gain in the detection system. One of the potential sources of inaccuracies might be the temperature instability of the voltage reference in combination with the temperature drift of integrated semiconductor components such as transistors and operational amplifiers within the ASIC chip. The complex combinations of these effects can cause a self-rising value of the applied threshold on the measurement (number of clock ticks) and thus lead to a detected drift of the measured energies towards lower values.

For future research, it is recommended to focus on the identification of the source of the detected distortions and minimise it, for example, by successive testing of a separate readout chip or simulating its temperature sensitivity in collaboration with the designers. The stability of the electronics, in addition to proposing a complex correction method to minimise the effect of temperature, could be another target.

The presented results show the importance of recording the temperature during the experiment and its impact on the overall result and accuracy of the measurements. It should be noted that some measurements under presented environmental conditions are outside the manufacturer’s stated operating temperature range but may occur in some of the more demanding applications, and detectors’ responses to environmental parameters may reveal different dependencies for different materials and thicknesses, which were not part of the study. Nevertheless, results indicate that TPX3 has potential not only for the laboratory but also in homeland security applications and space research.

## Figures and Tables

**Table 1:** List of radiation sources and target materials with their characteristic energies.

Symbol	Element	Energy
Cu	Copper	8,046 keV
Mo	Molybdenum	17,480 keV
In	Indium	24,209 keV
Ta	Tantalum	57,532 keV
^133^Ba	Barium	30.973 keV
		80,998 keV

## Data Availability

The data presented in this study are available from the corresponding author on request.
